# Carotenoid Distribution in Living Cells of *Haematococcus pluvialis* (Chlorophyceae)

**DOI:** 10.1371/journal.pone.0024302

**Published:** 2011-09-06

**Authors:** Aaron M. Collins, Howland D. T. Jones, Danxiang Han, Qiang Hu, Thomas E. Beechem, Jerilyn A. Timlin

**Affiliations:** 1 Department of Bioenergy and Defense Technologies, Sandia National Laboratories, Albuquerque, New Mexico, United States of America; 2 Department of Nanomaterials Sciences, Sandia National Laboratories, Albuquerque, New Mexico, United States of America; 3 Laboratory for Algae Research and Biotechnology, Department of Applied Sciences and Mathematics, Arizona State University, Mesa, Arizona, United States of America; US Dept. of Agriculture – Agricultural Research Service (USDA-ARS), United States of America

## Abstract

*Haematococcus pluvialis* is a freshwater unicellular green microalga belonging to the class Chlorophyceae and is of commercial interest for its ability to accumulate massive amounts of the red ketocarotenoid astaxanthin (3,3′-dihydroxy-β,β-carotene-4,4′-dione). Using confocal Raman microscopy and multivariate analysis, we demonstrate the ability to spectrally resolve resonance–enhanced Raman signatures associated with astaxanthin and β-carotene along with chlorophyll fluorescence. By mathematically isolating these spectral signatures, in turn, it is possible to locate these species independent of each other in living cells of *H*. *pluvialis* in various stages of the life cycle. Chlorophyll emission was found only in the chloroplast whereas astaxanthin was identified within globular and punctate regions of the cytoplasmic space. Moreover, we found evidence for β-carotene to be co-located with both the chloroplast and astaxanthin in the cytosol. These observations imply that β-carotene is a precursor for astaxanthin and the synthesis of astaxanthin occurs outside the chloroplast. Our work demonstrates the broad utility of confocal Raman microscopy to resolve spectral signatures of highly similar chromophores in living cells.

## Introduction

Carotenoids are tetraterpenoid pigments that are naturally occurring in all photosynthetic organisms, some bacteria and fungi as well as some animals. These pigments have a remarkable diversity of function. For example, in photosynthetic organisms, carotenoids can serve as light-harvesting molecules by filling the 400–500 nm gap where chlorophyll and bacteriochlorophyll do not appreciably absorb and may also serve a structural role in protein complexes. Furthermore, carotenoids have an important role in the dissipation of excess light intensities. For example, in plants and algae this is achieved through the xanthophyll cycle [1] and is part of non-photochemical quenching [2] of the excited state of chlorophyll. Carotenoids that are located in the chloroplast and essential for photosynthesis are called primary carotenoids.

Carotenoids that are neither located in the chloroplast of plants and algae nor are essential for photosynthesis are termed secondary carotenoids [3]. Many taxa of microalgae, such as *Chlorella zofingiensis, Dunaliella salina*, and *H. pluvialis* [4]–[7], have been reported to accumulate secondary carotenoids in lipid bodies under stress conditions. However, the molecular and cellular mechanisms for synthesis and accumulation of secondary carotenoids remains elusive. Most stress conditions generate reactive oxygen species (ROS) that can damage DNA, proteins, and membranes, while at the same time ROS triggers the production of secondary carotenoids that consume molecular oxygen during the synthesis process whereby reducing ROS formation [8]. The resulting secondary carotenoids also possess antioxidant activity that may quench ^1^O_2_ and and scavenge ROS as to mitigate the cytotoxic effect of oxidative damage [9], [10]. Moreover, secondary carotenogenesis is a useful way to store energy and carbon and helps to increase the cell survival under stressful conditions [6].

*H. pluvialis* is an ubiquitous freshwater green micoalga that is known to synthesize and accumulate esters of the red ketocarotenoid astaxanthin (3,3′-dihydroxy-β,β-carotene-4,4′-dione) under stress conditions such as: nutrient deprivation [11], increased salinity [8], [12], high irradiance [13], [14], and low/high temperature [15], as well as combinations of these stresses [6], [16]. Under stress conditions, β-carotene is speculated to be transported across the chloroplast membrane and is converted to astaxanthin, which is accumulated in lipid vesicles outside the chloroplast [17], [18]. However, direct evidence of this process in living cells is lacking. Astaxanthin can reach up to 4% of the total cellular dry weight [15], [17].

While progress has been made in describing the function of primary and secondary carotenoids from a variety of organisms, considerably less has been done to spatially resolve their locations *in vivo*. A suitable spectroscopic technique to identify carotenoids based on their molecular vibrations is Raman spectroscopy, and when paired with the spatial resolution of a confocal microscope, provides a powerful technique to address this question. Raman spectroscopy and microspectroscopy have recently been extended to algal research as they can provide molecular information with microscopic resolution, particularly for non-fluorescent biomolecules [19]. Researchers have successfully used Raman spectroscopy to investigate nutrient status [20], lipid profiling [21], [22], lipid identification and mapping [23], [24], and single carotenoid mapping [25]–[27]. In this work, we investigated carotenogenesis in living *H. pluvialis* cells using resonance-enhanced confocal Raman microscopy and multivariate curve resolution (MCR) analysis. In previous work, multivariate analysis and hyperspectral fluorescence microscopy were used to spectrally and spatially resolve highly-overlapping pigments in individual cyanobacterial cells [28] and here, we make an important extension of the MCR analysis to confocal Raman spectral images to resolve astaxanthin, β-carotene, and chlorophyll in living cells of *H. pluvialis*.

## Materials and Methods

### Culturing Conditions - *Haematococcus pluvialis*

SAG 34/1b was obtained from SAG culture collection at the University of Gottingen. Green flagellated cells of *H. pluvialis* were cultivated on Bold's Basal medium [29] that was modified to contain 3 times the nitrate source and vitamins (3N-BBM+V). Cultures were maintained on a platform shaker in baffled flasks. 50 µmol m^−2^ s^−1^ of cool-white fluorescent light was cycled on/off in 12-hour intervals, and the temperature was maintained between 24–27°C. Cells were also cultured in the predominantly non-motile palmelloid phase on modified BG-11 media [30]. Non-motile cells were maintained under the same growth conditions listed above however, the cells were manually shaken once a day. Cells were induced to accumulate astaxanthin, defined as inductive conditions, and form large red cysts (aplanospores) by centrifuging flagellated or palmelloid cells and resuspending the cells into BG-11 medium that was nitrate deplete. Cells were then exposed to continuous high-light (∼350 µmol m^−2^ s^−1^) for several days [31], [32].

### Spectroscopy of Purified Carotenoids

Astaxanthin and β-carotene were purchased from Sigma-Aldrich and lutein was purified from spinach leaves by HPLC according to a previously described method [33]. Stock solutions were prepared by dissolving each carotenoid in absolute ethanol and the concentrations were determined using the molar extinction coefficient of 125×10^3^, 145×10^3^ and 141×10^3^ M^−1^ cm^−1^ for astaxanthin, lutein and β-carotene, respectively [34]. For absorbance and Raman microscopy measurements, the stock carotenoids were diluted into pyridine. Bulk absorbance and fluorescence spectra were acquired on a Beckman DU640 spectrophotometer and a Jasco J-810 spectropolarimeter equipped with fluorescence detector, respectively. Raman spectra were acquired as described below.

### Confocal Raman Microscopy

Spectrally resolved images of *H. pluvialis* cells were acquired on a WiTec alpha3000R confocal Raman microscope. 532 nm laser excitation was coupled to the microscope through an optical fiber and directed to the sample through a 0.9 N.A. 100X coverslip-corrected objective. The power density measured at the sample was less than 1 mW. Raman scatter and fluorescence were collected by the same objective, focused through a 50 µm pinhole and dispersed by a 600 l/mm grating across an Andor Newton detector operated in EMCCD mode. The spectral resolution was not linear over the spectral range of the detector however, in the region of υ_1_ vibrational bands of carotenoids (∼1520 cm^−1^), the resolution was determined to be 1 cm^−1^. The resolution of the system was measured to be 0.47 µm (lateral) and 2.9 µm (axial).

Spectral images were acquired by raster scanning at approximately half the diffraction limited spatial resolution and integrating for times that ranged from 4–10 ms per spectrum. The time to acquire an image depended on the sample but was typically on the order of a few minutes. No sample degradation was observed with integrations times of 4–10 ms per pixel however, dwelling on a particular pixel for several seconds led to an exponential decrease (bleaching) in the Raman signal (result not shown). Bright-field images were taken before and after Raman acquisitions to ensure the integrity of the cells after imaging. Cells suspensions in the growth media listed above were directly loaded to a slide, covered with a glass coverslip. Motile cells were immobilized on 0.75% agar-coated slides. All cells remained hydrated for the duration of the Raman microscopy experiments.

### Multivariate Analysis

Raman spectral images were imported into Matlab (Mathworks Inc.) and converted into a data format that could be used by our internally developed, efficient and robust Matlab MCR software package described previously [35], [36]. A basic overview of the MCR algorithm can be found in [36] or an on-line theory and tutorial website (http://www.mcrals.info). MCR analysis has been described in detail elsewhere [37]–[41]. MCR is implemented using an iterative numerical analysis technique based on constrained alternating least square algorithm. The spectroscopic data are modeled by MCR by generating a basis set of spectral pure components with a corresponding set of concentrations that when combined together in a linear additive manner will reconstruct the spectroscopic data minus the noise and uncertainties. While we have used our patented software and algorithms for this work, MCR software packages are commercially available (e.g., Eigenvector Research, Inc., Wenatchee, WA, USA and CAMO, Inc., Woodbridge, NJ, USA ), Data from multiple spectral images (totaling 225,739 spectra) of each cellular morphotype were combined into a single composite image [28], spatially compressed by a factor of 4 to increase analysis speed and conserve memory [42], and our MCR algorithms were applied to yield a single set of 4 spectral components plus an offset that accounted for a least-squares fit of >99% for all spectral variance. This set of pure spectral components was then projected onto the uncompressed spectral images of the various morphotypes of *H. pluvialis* using classical least squares to generate the component concentrations for each pixel of the original image data set [43]. The mean intensities of MCR spectral components were calculated by masking off regions of the image were no cells were present and computing the average intensities for each individual component for the remaining pixels.

## Results

**Figure 1:**
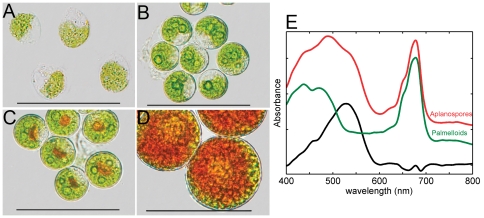
Transmission light microscopy images of cells of *H. pluvialis* in various growth stages. **A**. Bi-flagellated, motile cells. **B**. Green palmelloids under non-inductive conditions. **C**. Palmelloids under inductive conditions (high-light and nitrate deplete) for 24 hours. **D**. Predominantly red aplanospores under inductive conditions for 5 days. In all images, the scale bar represents 50 µm. **E**. Absorbance spectra of cells from the same cultures represented in **B** and **D**, palmelloids (green) and aplanospores (red), respectively, normalized to 1 at the peak of the chlorophyll absorption and the difference between the two spectra (black). Each trace has been offset by 0.2 au for clarity.

Transmission light microscopy verified that *H. pluvialis* cells were cultured as green bi-flagellates (Fig. 1A) or palmelloids (Fig. 1B) and induced to accumulate astaxanthin by stressing cells with nitrate-limitation and high-light irradiance. After 24 hours, red globular regions appeared towards the center of palmelloid cells (Fig. 1C), and after 5 days of the same treatment, cells had formed aplanospores, appearing mostly red and had significantly enlarged (Fig. 1D). The difference between the absorption spectra of green palmelloid cells and red aplanospores (Fig. 1E) shows a broad, band centered at 528 nm that represents the *in vivo* absorption of astaxanthin and closely resembles previously reported spectra [44].

**Figure 2:**
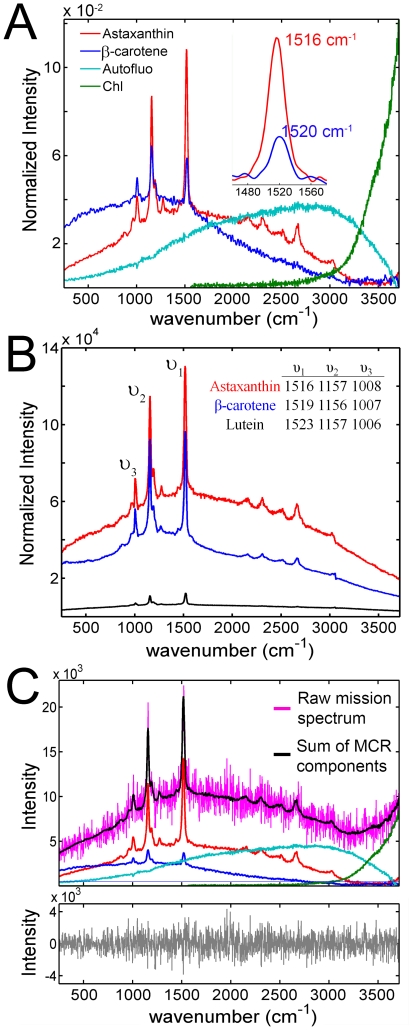
Multivariate analysis of confocal Raman spectral images. **A**. MCR derived, pure spectral components. 225,739 spectra, representing 19 spectral images of *H. pluvialis* cells in all three cellular morphotypes were combined into a single dataset and analyzed using a constrained iterative MCR algorithm. **B**. 532 nm – excited Raman spectra of pure astaxanthin (red), β-carotene (blue), and lutein (black) dissolved in pyridine. The data are normalized to represent equivalent molarities of each carotenoid under identical acquisition parameters. The positions of the υ_1_, υ_2_, υ_3_, bands are indicated in the inset table in units of cm^−1^. Raman band contributions of pyridine have been subtracted from each spectrum. **C** *upper trace* - Raw spectrum (pink) of an individual pixel from a palmelloid cell type and the MCR modeled fit (black) which is the linear sum of spectral components indicated below the fit and colored according to **A**. **C** *lower trace* –difference between the raw spectrum and MCR model on an expanded y scale.

Cells of *H. pluvialis*, under the same conditions mentioned above were investigated using Raman microscopy with 532 nm laser excitation and the resulting spectral images were analyzed with MCR. MCR extracts the independently varying pure spectral components *a priori* without the need for reference data representing expected chemical species. Additionally, the method also deduces independent concentration maps that detail the location and relative abundance of each spectral component. From the analysis, four independently varying spectral components were derived from our examinations of the *H. pluvialis* morphotypes. Specifically, two Raman signatures superimposed with fluorescence characteristic of carotenoids were identified in combination with broad, featureless fluorescence bands likely originating from cellular autofluorescence and fluorescence emission attributed to chlorophyll (Fig. 2A). MCR algorithms successfully extract highly-overlapping spectral features if the intensity of each component varies across spatial pixels. In other words, the relative concentration of each species must differ throughout the image.

Carotenoids possess three intense Raman scattering modes and are defined as; υ_1_ – conjugated C  =  C stretching vibrations, υ_2_ – C-C vibrations coupled to C-CH_3_ stretches or C-H in-plane bending, and υ_3_ – CH_3_ stretching modes [45]. Of these three bands, υ_1_ is the most diagnostic for carotenoid identity and the υ_1_ Raman bands for the two carotenoid pure spectral components were centered at 1516 cm^−1^ and 1520 cm^−1^ (Fig. 2A-inset).

To confirm the MCR derived carotenoid identities, resonance enhanced Raman spectra were acquired for pure astaxanthin, β-carotene, and lutein dissolved in pyridine (Fig. 2B) as these are the most abundant carotenoids found in cells of *H. pluvialis*. The polarizability of pyridine (*n* = 1.5092) has been found to mimic the environment of membranes and lipids [46]. The data are normalized to represent the same molarity and acquisition parameters. The υ_1_ bands for pure lutein, β-carotene and astaxanthin were centered at 1523 cm^−1^, 1519 cm^−1^ and 1516 cm^−1^, respectively. Lutein is the dominant carotenoid in the chloroplast, accounting for 50–60% of the total carotenoid content in green cells [47], [48]. However, the resonance Raman effect is expected to scale as the square of the absorption probability at the excitation wavelength (532 nm) and lutein does not absorb appreciably compared to astaxanthin and β-carotene (Fig. S1). Moreover, other primary carotenoids such as neoxanthin and violaxanthin, that are associated with light-harvesting complex II, have absorption spectra (in pyridine) blue-shifted compared to lutein [46] and the resonance effect is also expected to be insignificant. We therefore assign the Raman components of the MCR model (Fig. 2A) with υ_1_ = 1516 cm^−1^ as astaxanthin and υ_1_ = 1520 cm^−1^ as β-carotene. This assignment is corroborated by their respective cellular location as described below. The spectral component attributed to chlorophyll fluorescence shows only the blue edge of this feature as the spectrometer used in this analysis has a maximum spectral range of about 664 nm, which is approximately 20 nm away from the peak fluorescence intensity. Bulk fluorescence from cells of *H. pluvialis* is shown in supplemental Fig. S2 and supports this assignment. The MCR component attributed to autofluorescence is broad and featureless with a maximum around 630 nm. The feature is commonly observed in live cell imaging and likely originates from cellular flavins, NADPH, mucilage, and other unknown sources of emission [49], [50].

The fit of these MCR-derived spectral components is shown for a representative single pixel spectrum from a palmelloid cell under inductive conditions (Fig. 2C, *upper trace*), and the excellent quality of the MCR model is verified by the structureless character of the unmodeled residuals (Fig. 2C, *lower trace*).

**Figure 3:**
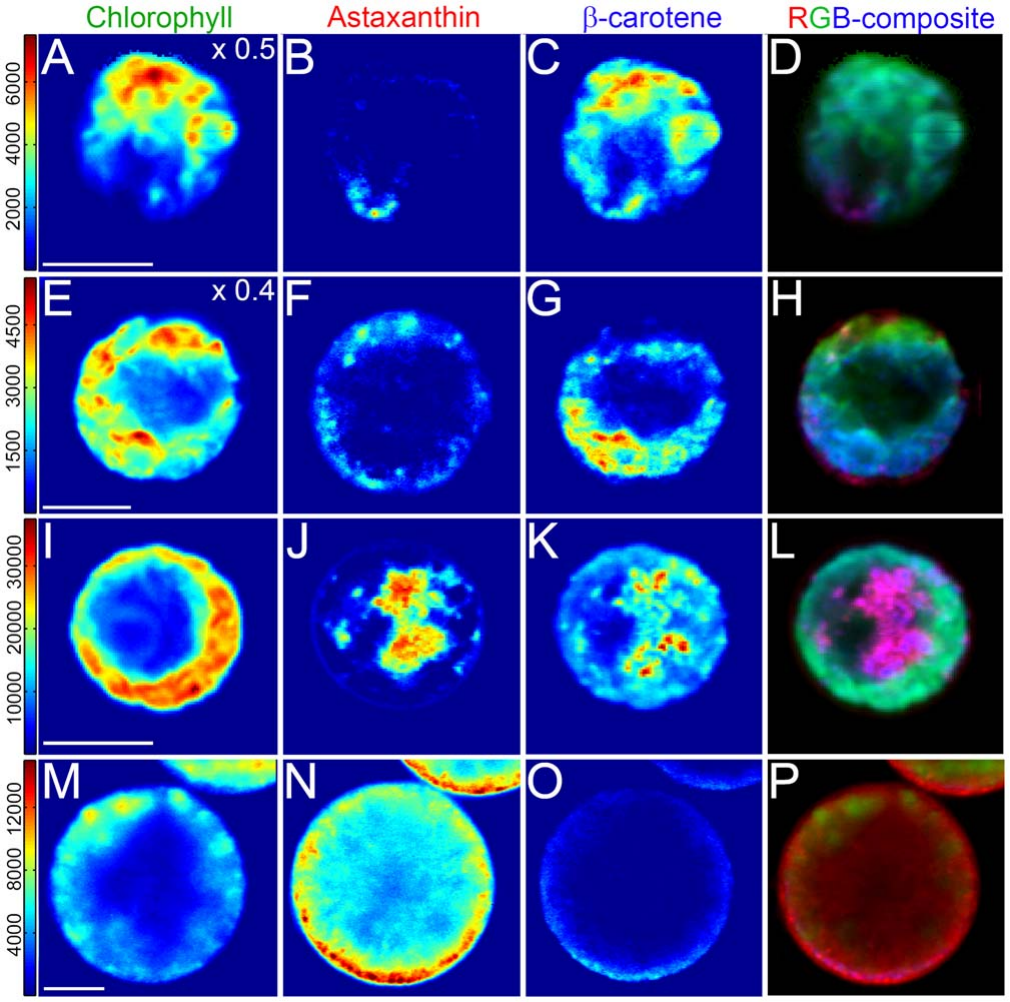
MCR concentration maps for spectral components representing chlorophyll, astaxanthin, and β-carotene. **A–D**. Flagellated motile cell. **E–H**. Palmella stage cell under non-inductive conditions **I–L**. Palmelloid cell under inductive conditions for 24 hours and **M–P**. Large red cyst (aplanospore). In all images the scale bar represents 10 µm. Note that in **A** and **E**, the intensity has been scaled by 0.5 and 0.4, respectively for clarity. The intensity scales are not comparable across cell types as the acquisition parameters were optimized for each cell image however, within a cell type the intensities within the concentration map represent relative component concentrations. Composite RGB images are created by overlaying the individual concentration maps that have been pseudo-colored as indicated in the image titles.

The MCR model was then applied to each spectrum of the original images to reconstruct the location and relative concentration of each component at every spatial pixel. Fig 3A–P shows representative concentration maps for astaxanthin, β-carotene and chlorophyll as well as composite red-green-blue images that deconvolute the cellular locations of these chromophores. In flagellated (Fig. 3A–D) and palmelloid cell (Fig. 3E–H) types under non-inductive conditions, chlorophyll is positioned only in the chloroplast and the overall distribution follows its cellular location as assessed by transmission light microscopy (Fig. 1A and B). β-carotene was co-localized in the chloroplast although its intensity was ∼2x less than that of chlorophyll. For both of these morphotypes, astaxanthin is nearly undetectable. Faint traces of astaxanthin appear in the represented palmelloid cell toward the cell periphery (Fig 3H) however, its intensity was weak.

After 24 hours of nitrogen limitation and high-light stress, cells began to accumulate astaxanthin as indicated in Fig. 3I–L. Again, chlorophyll is only observed in the chloroplast region. Interestingly, β-carotene is not only detected in regions of chlorophyll emission but also high concentrations of β-carotene are found outside of the chloroplast, co-localized with astaxanthin in globular and punctate regions. These results are consistent with the hypothesis that β-carotene is a precursor in the biosynthesis of astaxanthin and that this conversion takes place outside of the chloroplast and in lipid vesicles, [17], [51] and provide the first experimental evidence for this hypothesis. Astaxanthin intensity was found concentrated towards the cell center and was completely devoid in chlorophyll containing regions. The composite image from the concentration maps emphasizes the cellular location of these components (Fig 3L).

**Figure 4:**
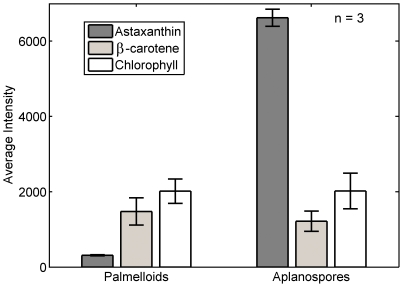
Comparison of the pigment concentrations between flagellates and aplanospores. The mean intensities for each spectral component were calculated for each cell type on a per cell basis. The standard deviations are indicated by error bars. Further details on how the image means were calculated are presented in the *Material and Methods*.

Finally, after 5 days of the same stress treatment, palmelloid cells accumulating astaxanthin have transformed into resting aplanospores (Fig. 3M–P). Chlorophyll intensity is weakly observed towards the cell periphery along with β-carotene that appeared diminished. The concentration of astaxanthin was significantly increased throughout the entire cell. Comparison of the relative concentrations of pigments between flagellate and aplanospore cell types on a per cell basis was assessed by comparing the cell image mean intensities (Note: These data were obtained under identical acquisition parameters enabling this comparison to be made.). The mean intensity of astaxanthin shows a dramatic increase for aplanospore cell types while β-carotene and chlorophyll are essentially unchanged (Fig. 4). Astaxanthin appeared more concentrated at the periphery of aplanospores where cytosolic lipid vesicles are located in these cell types [18].

## Discussion

We have used confocal Raman microscopy and MCR analysis to extract multiple pure spectral components from a complex dataset containing resonance-enhanced Raman and fluorescence features while also spatially mapping the changes in pigment composition in different cellular morphotypes of *H. pluvialis*. MCR algorithms resolve overlapping spectral components over the entire spectral range and have a distinct advantage over simple band integration that only considers a narrow spectral window as is demonstrated in Fig. S3. In this example, the Raman signatures are convoluted with fluorescence making their true cellular locations ambiguous using band integration. Furthermore, weak spectral features will be “buried” underneath dominant components and might be obscured or missed entirely. MCR algorithms however, analyze spectral image datasets without the need for laborious background subtraction of fluorescence and other non-Raman sources. Actually, by modeling the combination of fluorescence and Raman spectral features we observe improved spectral resolving power. For example, the intensity of Raman bands ascribed to β-carotene is about ∼0.25 times as intense as those of astaxanthin (Fig. 2A) however, because MCR generates a spectral component that includes the weak fluorescence from this same species, the delineation is more obvious.

The ability to reliably spectrally and spatially resolve multiple chromophores in living cells provides a unique opportunity to probe carotenogenesis *in vivo*. For example, an important enzyme in the conversion of β-carotene into astaxanthin is β-carotene-C4-oxygenase (for a comprehensive review of synthesis of secondary carotenoids in *H. pluvialis* see [6]). Inhibition of this enzyme with diphenylamine resulted in the accumulation of β-carotene in lipid bodies outside of the chloroplast [47], [48]. Moreover, it was also reported that β-carotene was transported across the chloroplast membrane [51] and that astaxanthin synthesis occurs in lipid vesicles [17]. Indeed, we find evidence for considerable β-carotene accretion in the cytosol in distinct regions and co-localized with astaxanthin (rather than chlorophyll) after 24 hours of stress conditions (Fig. 3L) providing *in vivo* evidence that the site of β-carotene conversion must occur, at least partly, outside the chloroplast and presumably in lipid vesicles. Pigment quantification of cell extracts have indicated that chlorophyll and β-carotene concentrations are essentially stable during astaxanthin accumulation when cells transform from early flagellate cells into large, red aplanospores [52]. By determining the mean cellular intensities for chlorophyll, astaxanthin and β-carotene in flagellate vs. aplanospore cells we are able to determine the relative concentrations of each component at the single-cell level during these vastly different stages of cell growth. Our results calculated at the single-cell level are consistent with these bulk pigment measurements (Fig. 4), however the detailed spatial information provided by Raman spectral images permits the visualization of pigment location unavailable with bulk pigment measurement techniques.

Additional excitation sources would extend the utility of confocal Raman microscopy. For example, a shorter wavelength laser could be sensitive to additional carotenoids such as lutein and even though the cellular location of lutein is expected to overlap with β-carotene in the chloroplast, its isolation and analysis by MCR could provide relative quantification information. Conversely, a longer wavelength excitation source would likely not result in resonance enhancement of the Raman signal for carotenoids, it could however, avoid contaminating and often overwhelming fluorescence.

We have presented the first use of Raman microscopy and multivariate analysis to extract multiple, spectrally and spatially over-lapping chromophores and resolve their spatial locations in living algal cells. Such approaches should be applicable to a wide range of complex and dynamic biological systems.

## Supporting Information

Figure S1 **Absorption spectra of pure carotenoids in pyridine.** Astaxanthin (red), β-carotene (blue) and Lutein (black) have been normalized to their respective absorption maxima. The laser excitation (532 nm) wavelength is indicated by the vertical dashed line.
(TIF)

Figure S2 **Bulk fluorescence from green cells of** ***H. pluvialis*** **.** The emission maximum was at 685 nm. The inset shows the same spectrum on the spectral range of the spectrometer used on the Raman microscope.
(TIF)

Figure S3 **Comparison of multivariate analysis to band integration.** *Upper trace* – palmelloid cell under inductive conditions analyzed using the MCR-derived model indicated in Fig. 2A. The components are; astaxanthin (**A**), chlorophyll (**B**) and β-carotene (**C**). The composite RGB image is shown in **D**. *Lower trace* – the same spectral image analyzed by integrating the area under diagnostic bands for each species. The 1514–1517 cm^−1^ (**E**) and 1518–1521 cm^−1^ (**G**) integrated images should partially capture the υ_1_ Raman mode of astaxanthin and β-carotene, respectively and the 3200–3700 cm^−1^ (**F**) integrated image should represent chlorophyll emission. The RGB-composite of these three images is shown in **H**. The scale bar for both images presents 10 µm.
(TIF)
