# Supplementary figures and images for: Carotenoid Distribution in Living Cells of Haematococcus pluvialis (Chlorophyceae)
Source: PLoS One. 2011 Sep 6;6(9):e24302. doi: 10.1371/journal.pone.0024302 (PMC3167842; doi:10.1371/journal.pone.0024302)

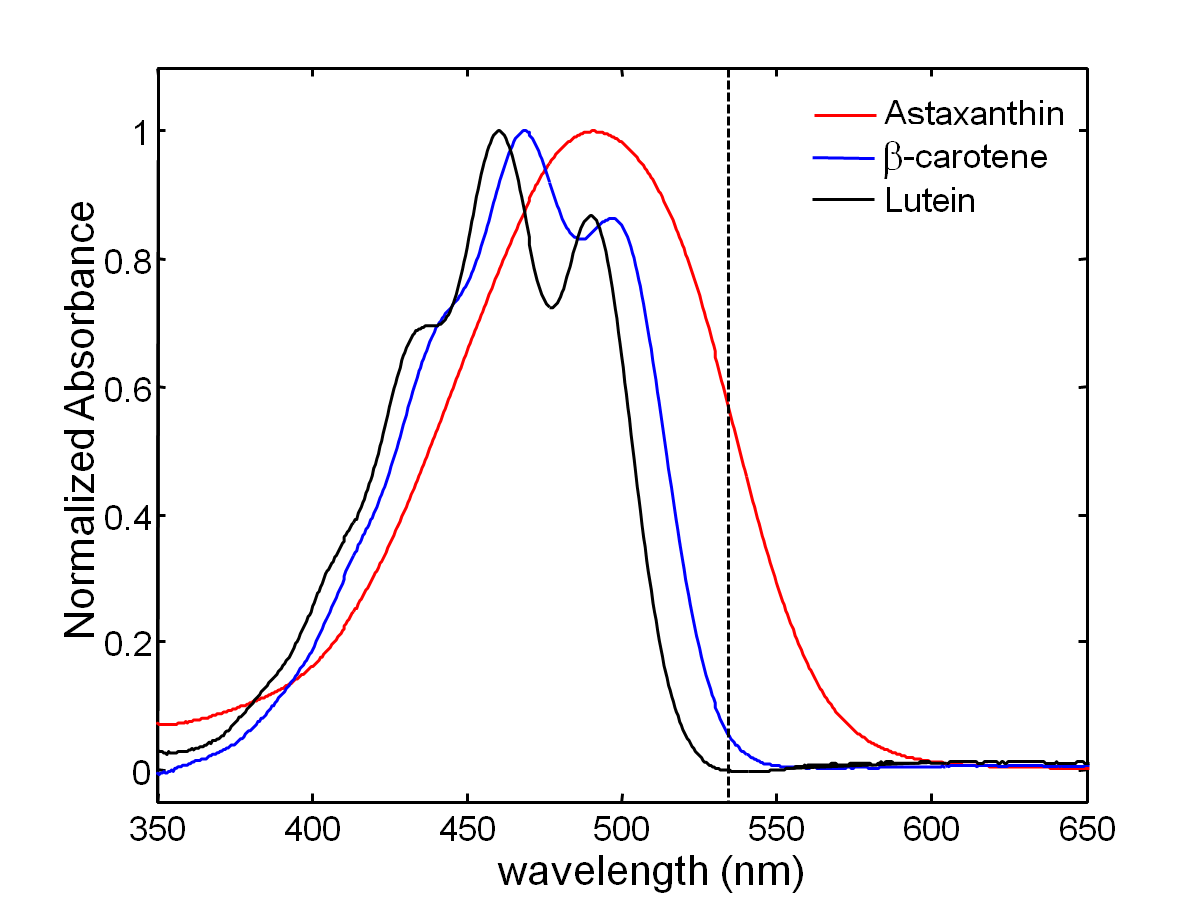

Supplement: Figure S1 — Absorption spectra of pure carotenoids in pyridine. Astaxanthin (red), β-carotene (blue) and Lutein (black) have been normalized to their respective absorption maxima. The laser excitation (532 nm) wavelength is indicated by the vertical dashed line. (TIF) [file pone.0024302.s001.tif]

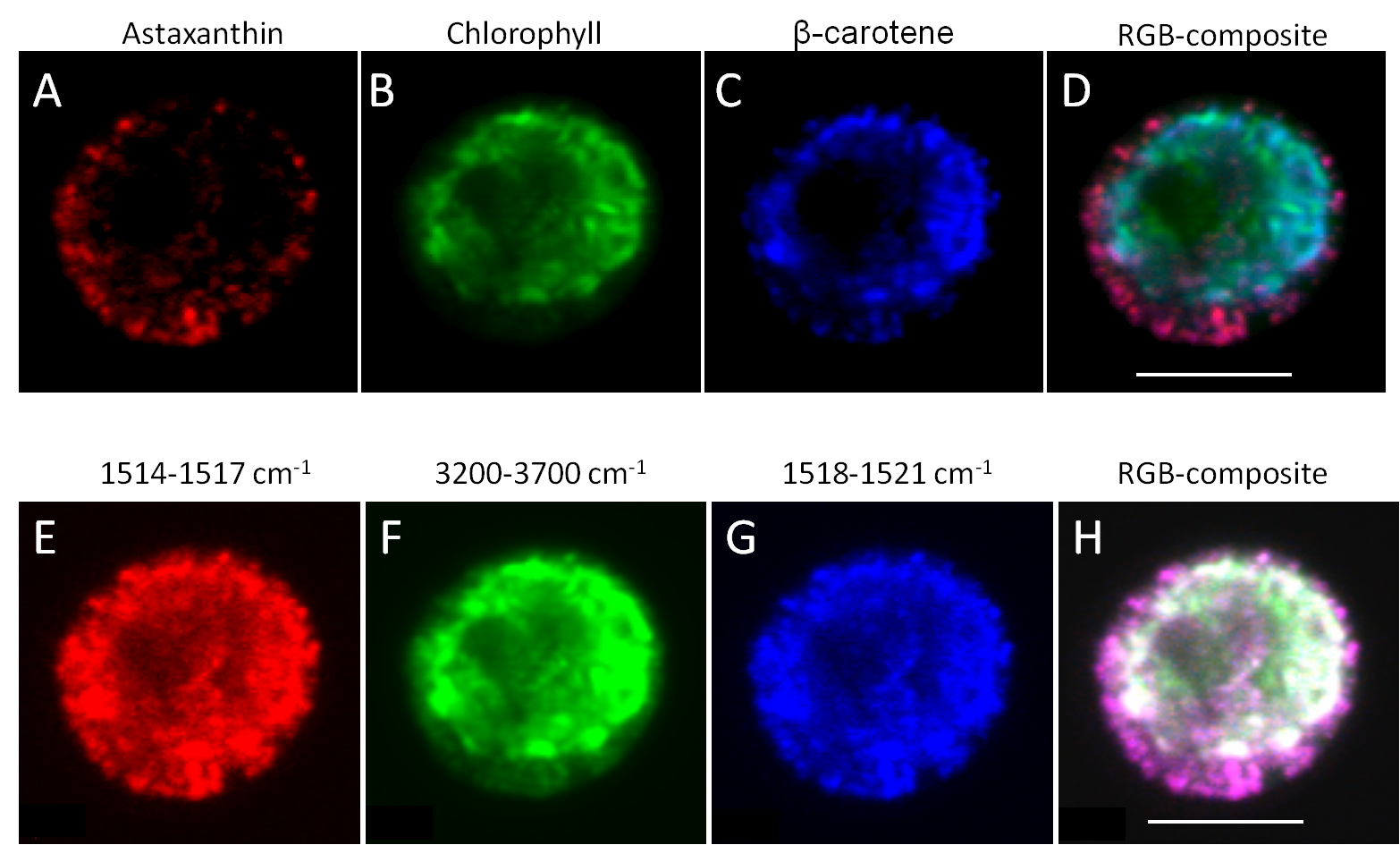

Supplement: Figure S3 — Comparison of multivariate analysis to band integration. Upper trace – palmelloid cell under inductive conditions analyzed using the MCR-derived model indicated in Fig. 2A. The components are; astaxanthin (A), chlorophyll (B) and β-carotene (C). The composite RGB image is shown in D. Lower trace – the same spectral image analyzed by integrating the area under diagnostic bands for each species. The 1514–1517 cm−1 (E) and 1518–1521 cm−1 (G) integrated images should partially capture the υ1 Raman mode of astaxanthin and β-carotene, respectively and the 3200–3700 cm−1 (F) integrated image should represent chlorophyll emission. The RGB-composite of these three images is shown in H. The scale bar for both images presents 10 µm. (TIF) [file pone.0024302.s003.tif]
